# Sex Differences in Prognostic Markers: Exploring Outcome Variability After Mechanical Thrombectomy in Large Vessel Occlusion Stroke

**DOI:** 10.3390/jcm14217855

**Published:** 2025-11-05

**Authors:** Hannes Schacht, Alexander Neumann, Nora Petersen, Lis Merrit Ehm, Maria Marburg, Christine Matthis, Ulf Jensen-Kondering, Peter Schramm, Jens Minnerup, Georg Royl, Philipp J. Koch

**Affiliations:** 1 Department of Neuroradiology, University Hospital Schleswig-Holstein, Campus Lübeck, 23538 Lübeck, Germany; 2 Department of Neurology, University Hospital Schleswig-Holstein, Campus Lübeck, 23538 Lübeck, Germany; 3 Department of Social Medicine and Epidemiology, University Hospital Schleswig-Holstein, Campus Lübeck, 23538 Lübeck, Germany; 4 Department of Neurology and Experimental Neurology, Charité—University Medicine Berlin, Corporate Member of Freie Universität Berlin and Humboldt-Universität zu Berlin, 10117 Berlin, Germany

**Keywords:** stroke, large vessel occlusion, thrombectomy, perfusion imaging

## Abstract

**Background:** Sex-related disparities in long-term outcomes after large vessel occlusion (LVO) following mechanical thrombectomy (MT) have been repeatedly shown. Notably, a lower likelihood of achieving functional independence 90 days post-stroke has been found in women. However, most studies showed equal outcomes for both sexes after MT. It remains unclear whether there are sex differences in the prognostic values of clinical and neuroradiological parameters. Our investigation aimed to discern the divergent prognostic values of multiple markers between sexes. **Methods:** We retrospectively examined 183 stroke patients with LVO who received MT. Using multivariable logistic regression models, we investigated sex-specific associations of various parameters, including ASPECTS, lesion core volume, penumbra volume, collateral status, and time to reperfusion, concerning outcomes at discharge and 90 days post-stroke. **Results:** We observed no significant difference between men and women in achieving a favorable outcome defined as modified Rankin Scale (mRS) 0–2. However, when considering the full mRS, women exhibited less favorable overall outcomes. In women, NIHSS, TICI score, and penumbra volume were associated with outcome, whereas in men, core lesion volume and ASPECTS were associated. Age was the only factor associated with outcome in both sexes. **Conclusions:** Considering the full spectrum of mRS may provide more sophisticated understanding of sex-related outcome differences. Further, these findings highlight the importance sex-specific prognostic factors in outcome assessment. Unraveling sex-specific prognostic biomarkers of recovery has the potential to advance precision medicine and personalized clinical management in stroke.

## 1. Introduction

Despite significant success in randomized controlled trials (RCT) assessing the group benefit of endovascular treatment for large vessel occlusion (LVO), expanding the selection criteria to include extended time windows and larger lesion core volumes presents a major challenge in the clinical management of acute stroke due to the considerable variability in outcomes. While endovascular therapy demonstrates a strong therapeutic effect on a group level, a large proportion of patients remain severely disabled, unable to walk or bedridden, and entirely dependent on external assistance and nursing care. Identifying potential markers linked to functional outcome and therapeutic benefits may improve existing predictive modeling, allowing for more accurate prediction of individual functional outcomes. This is essential for discussing therapeutic options and prognosis with patients and their relatives. Furthermore, personalized predictive modeling may ultimately aid clinical management and therapeutic decisions, moving acute stroke therapy towards a more individualized, personalized approach.

In this context, several clinical and imaging parameters have been identified to be associated with functional outcome. Specifically, older age, the severity of initial deficits, and the time elapsed between imaging and reperfusion appear to be the prominent clinical markers for outcome variability [1,2,3,4,5]. Additional analyses of the initial neuroimaging findings have been introduced to assess prognostic value. Measurements such as core lesion volume, mismatch profile, collateral status, lesion water uptake, involvement of specific Alberta stroke program early CT score (ASPECTS) regions and the involvement of the corticospinal tract and dopaminergic networks were found to correlate with functional outcome [5,6,7,8,9,10,11]. There is disagreement in the literature regarding whether there is a different likelihood of a favorable outcome between males and females. While several studies indicate a higher benefit in males [12,13,14,15,16,17,18,19], most pooled data, meta-analyses, and real-world findings do not reveal any differences [1,20,21,22,23,24,25,26,27,28]. However, it remains uncertain whether clinical and imaging prognostic markers are equally relevant in both sexes. Identifying sex-specific differences in prognostic markers could significantly improve predictive models for functional outcomes following mechanical thrombectomy (MT). In a retrospective analysis, we examined sex-dependent associations of known prognostic markers with functional outcome in a group of patients with LVO of the anterior circulation who underwent MT.

## 2. Materials and Methods

### 2.1. Patient Selection

In a single center retrospective analysis, patients who were admitted from August 2014 to April 2020 with anterior circulation stroke due to LVO and within a maximum of six hours after symptom onset were included. Inclusion criteria were (i) CT-angiography confirming LVO of the anterior circulation, (ii) MT, (iii) full assessment of the National Institutes of Health Stroke Scale (NIHSS) at admission and the modified Rankin Scale (mRS) at discharge and after 90 days. Isolated occlusions of peripheral branches (A2, M2 and more distal) were excluded.

### 2.2. Image Acquisition and Analysis

Non-enhanced cranial CT images (NCCT), as well as supra-aortic CT angiography and CT perfusion examinations, were acquired on two different scanners (Siemens Somatom Definition AS, Siemens Healthineers, Erlangen, Germany and Philips CT 6000 iCT, Philips Healthcare, Hamburg, Germany). Cerebral blood flow (CBF) and time-to-maximum of the tissue residue function (Tmax) maps were derived from perfusion images with a 5.0 mm slice thickness with syngo.via (Siemens Healthcare, Forchheim, Germany). Tissue at risk was defined as Tmax > 6 s. The core lesion volume was estimated by CBF < 30% of the mean of the healthy hemisphere, following previous works [29,30,31]. The core lesion volume was subtracted from the volume of the tissue at risk to calculate the penumbra volume. Volumes were set in relation to the volume of the whole brain. Calculations were performed using Python (v.3.9.13). Based on CT angiography images, the collateral status was assessed using a modified Miteff system (good: contrast filling within the entire middle cerebral artery (MCA) distal to the occluded segment; moderate: only partial contrast filling of the MCA distal to the occluded segment, with some MCA branches visible in the Sylvian fissure; poor: contrast filling only in superficial MCA branches) [32]. The scoring was performed by an experienced neuroradiologist who was blinded to the clinical scores. The ASPECTS ratings of the NCCT images were performed by two experienced neuroradiologists, blinded to the clinical scores. In case of disagreement between the two raters, the median was taken.

### 2.3. Clinical and Demographic Data

The primary clinical outcome measure was defined as favorable outcome, represented by an mRS score from 0 to 2, assessed both at discharge and 90 days after stroke. In a secondary analysis, the complete ordinal spectrum of the mRS was utilized as an outcome. Additional clinical parameters included age, NIHSS at admission, sex, the Thrombolysis in Cerebral Infarction (TICI) score, the time between symptom onset and admission, and the time between imaging and reperfusion. All this data was extracted from the medical charts.

### 2.4. Statistical Analysis

Clinical parameters were tested for sex differences using either Student’s *t*-test or Mann–Whitney U test, as appropriate. The association between sex and both favorable outcome and the spectrum of the mRS at discharge, as well as after 90 days, was assessed using multivariable ordinal logistic regression models, fitting odds ratios (OR) with 95% confidence intervals (CI) to an increase in mRS while adjusting for age, ASPECTS, NIHSS at admission, and TICI score.

To evaluate the sex-dependent influence of prognostic markers on functional outcomes after MT, we tested the impact of potential prognostic markers on functional outcome using multivariable logistic regression models for males and females independently. All models were adjusted for the effects of age, NIHSS at onset, ASPECTS, and TICI score. For lesion core volume, ASPECTS was excluded as an additional covariable due to high intercorrelation. Sensitivity analyses were performed adjusting for the influence of atrial fibrillation. Statistical analyses were conducted using Python (v. 3.9.13; Toolboxes used: SciPy, statsmodels). Statistical significance was assumed for *p*-values < 0.05.

## 3. Results

### 3.1. Clinical and Demographic Data

A total of 183 patients with complete clinical data were included in the final analyses. Comparisons of clinical and demographic characteristics are reported in Table 1. Women were significantly older than men (75 vs. 70 years, *p* = 0.005). Additionally, women exhibited a higher incidence of atrial fibrillation (*p* = 0.021).

### 3.2. Association Between Sex and Functional Outcome

There were no sex-specific treatment effects when adjusted for age, NIHSS, ASPECTS, and TICI score for favorable outcome (mRS < 2) at discharge (sex: OR: 1.29 (0.76–2.18), *p* = 0.343), as well as 90 days after stroke (sex: OR: 1.52 (1.00–2.31), *p* = 0.052).

**Figure 1:**
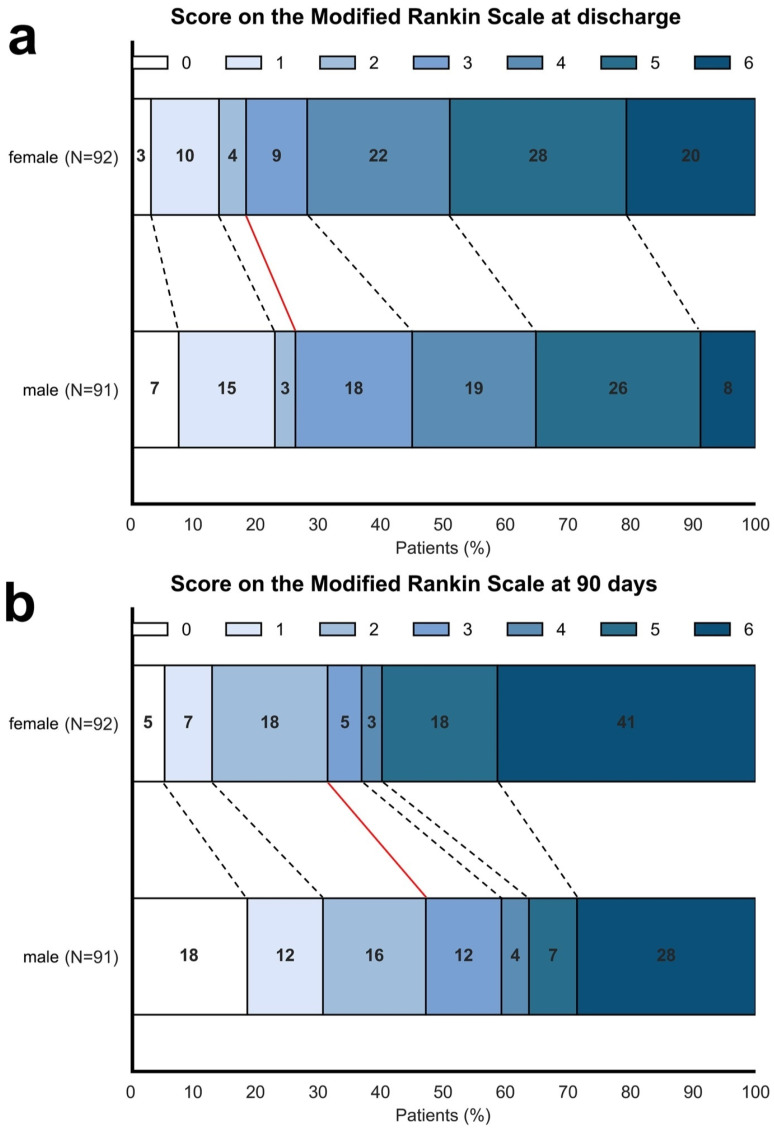
Shift plots illustrate the overall differences between female and male patients in the modified Rankin Scale (mRS) at discharge (**a**) and 90 days following stroke (**b**). Numbers represent the percentage of patients with the corresponding mRS score. Red lines mark the boundaries between favorable and unfavorable outcomes.

However, when considering the entire ordinal spectrum of the mRS, sex was associated with mRS at discharge, with an odds ratio of 0.47 (0.27–0.81, *p* = 0.006) favoring males, adjusted for age, NIHSS at onset, ASPECTS, and TICI score. Shift plots indicate a higher proportion of patients with favorable outcomes at discharge among males (25%) compared to females (17%), while 48% of female patients had an mRS of 5 and 6, versus 34% in males (Figure 1a).

Similarly, sex was associated with mRS 90 days after stroke, with an odds ratio of 0.43 (0.25–0.75, *p* = 0.003) favoring males, adjusted for age, NIHSS at onset, ASPECTS, and TICI score. The shift plot in Figure 1b illustrates a higher proportion of patients with favorable outcomes after 90 days among males (46%) compared to females (30%). Meanwhile, 59% of female patients scored mRS 5 and 6, in contrast to 35% of males (Figure 1b). The association between sex and functional outcome is additionally presented as supplementary online material in bar graphs (Figure S1).

Sex did not impact the changes in mRS from discharge to 90 days post-stroke, after adjusting for age, NIHSS at onset, ASPECTS, and TICI score (OR: 1.45 (0.83–2.52), *p* = 0.187).

### 3.3. Sex Dependent Associations of Prognostic Markers with Outcome

**Figure 2:**
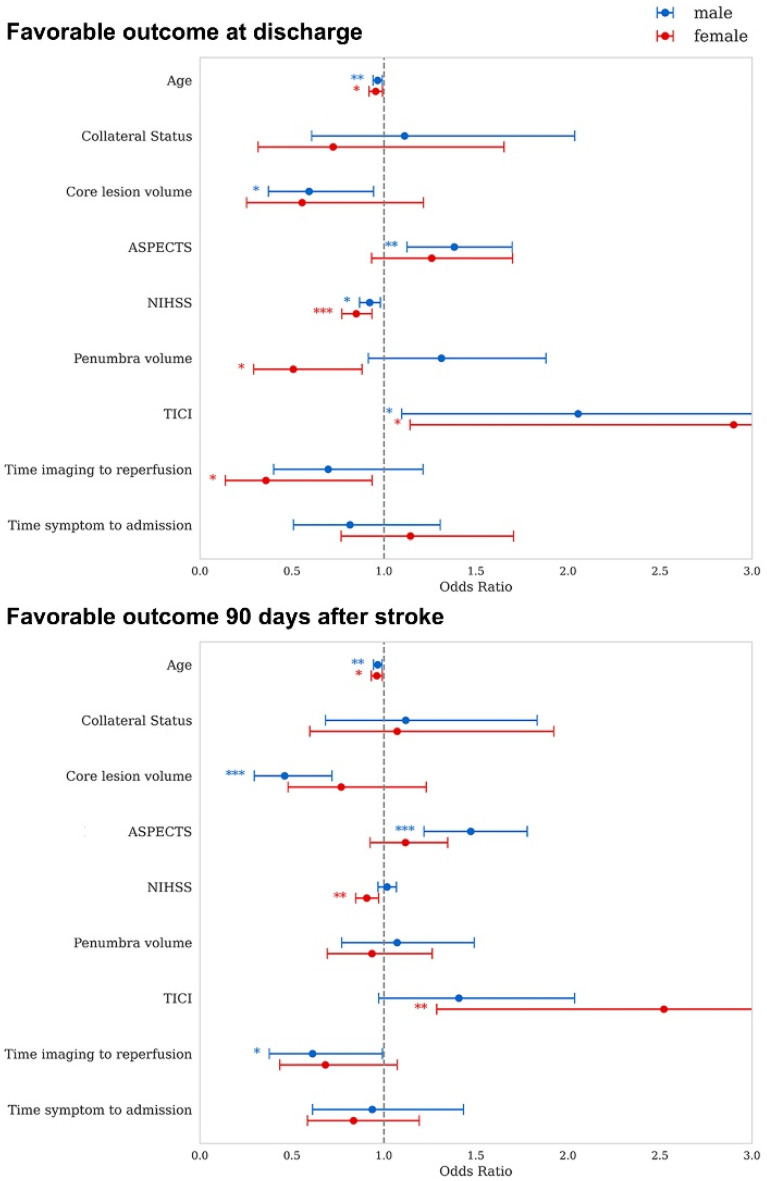
Forest plots showing sex-dependent associations of prognostic markers and outcome. Odds Ratios above 1 indicate that higher values are linked to a greater likelihood of favorable outcome. Asterisks mark significant associations (* *p* < 0.05; ** *p* < 0.01; *** *p* < 0.001). Red lines indicate the odds ratio and statistical significance of the respective prognostic marker for women; for men, these are shown in blue.

Significant differences were observed between female and male patients regarding prognostic markers that lead to favorable outcomes, both at discharge and 90 days post-stroke. Measurements of core lesion extent, namely ASPECTS and core lesion volume, demonstrated an association with favorable outcomes at discharge and 90 days following stroke, but only in men, where more extensive lesion extent was associated with less favorable outcome. In women, prognostic markers that were significantly associated with favorable outcomes included the NIHSS at admission and the TICI score, for both early and long-term outcomes. Lower NIHSS values and higher levels of revascularization were associated with better outcome. Higher penumbral volume was associated with worse functional outcome in women. In men, NIHSS and TICI score were associated with outcome only at discharge. Age was associated with both short- and long-term outcomes, independent of sex. Penumbra volume was associated only with short-term outcome in women. The time from imaging to reperfusion displayed a divergent association in relation to time and sex, regarding short-term outcome for women and long-term outcome for men. Figure 2 illustrates sex-dependent associations of prognostic markers and outcomes. Adjusting statistical models for the influence of atrial fibrillation did not alter the findings.

## 4. Discussion

### 4.1. Study Aim and Key Findings

In this retrospective analysis of clinical data, we demonstrated no significant sex differences in the likelihood of achieving a favorable outcome (mRS 0–2) after MT for LVO. However, when evaluating the full range of mRS scores, women exhibited less favorable overall outcomes. Notably, we identified distinct sex-specific patterns in the prognostic value of several clinical and imaging markers. In female patients, favorable outcomes were significantly associated with baseline NIHSS, TICI score and penumbra volume, whereas in male patients, core lesion metrics were predictive of favorable outcomes. Age emerged as the only prognostic marker consistently associated with both short- and long-term outcomes across sexes. These findings underscore the importance of incorporating sex-specific considerations when interpreting prognostic markers and developing predictive models for thrombectomy outcomes.

### 4.2. Association Between Sex and Functional Outcome

In our analyses, we observed no significant sex differences in the rate of favorable functional outcomes (mRS 0–2) following MT for LVO, consistent with findings from multiple studies, including RCTs, real-world cohort studies, and meta-analyses [1,20,21,22,23,24,25,26,27,28,33]. These studies largely report comparable outcomes between men and women after adjusting for relevant covariables. However, this apparent equivalence becomes more nuanced when examining the entire mRS distribution. In our data, women showed worse outcomes across the full mRS range—both at discharge and at 90 days—highlighting a sex-related disparity that is not captured when focusing solely on dichotomized outcomes. Several prior studies have similarly reported poorer outcomes in women [12,13,14,15,16,17,18,19], despite some evidence suggesting more favorable cerebral hemodynamics in female patients, which was observed by Lagebrant et al. and Wróbel et al. [17,19]. Notably, many of these studies include a relatively small proportion of female patients [13,18], a limitation that is also present in some studies reporting no sex differences [24,27]. Conversely, reports of superior outcomes in women are rare. For instance, Casetta et al. found higher rates of favorable outcomes in women, hypothesizing a potential benefit from intravenous thrombolysis additional to MT [34]. Demeestere et al. reported similar rates of favorable outcomes between the sexes but a lower frequency of poor outcomes among women when considering the full mRS spectrum [35]. Sun et al. observed better outcomes in women within the subgroup of patients with lower stroke severity (NIHSS < 15) [27].

As in previous studies, women in our cohort were significantly older than men—a baseline characteristic commonly cited as a potential contributor to sex differences in outcome [14,16,18,19,21,22,23,24,25,26,28,33,34,35]. In addition to the influence of age on the recovery potential after a stroke on its own, age may also play a role in the pre-hospital phase. Studies on sex-related differences in myocardial infarction showed that women live longer than their husbands, and are therefore more likely to live alone at symptom onset, so this is also conceivable in stroke [36]. While age was a significant predictor of outcome in our data, we did not observe an interaction between age and sex that would explain the observed disparities. Additionally, we observed an increased incidence of atrial fibrillation in women, which was also found in several other studies [24,25,28,34]. Nevertheless, this did not influence functional outcome nor did it modify the sex-dependent prognostic associations observed after mechanical thrombectomy.

Our findings support earlier reports indicating that female sex may be a potential risk factor for less favorable outcomes following MT for anterior circulation LVO. These findings emphasize the limitations of dichotomized outcome measures in capturing the full spectrum of sex-related differences in post-thrombectomy recovery. Moreover, they highlight the importance of distinguishing between data derived from highly selected RCT populations and more heterogeneous real-world cohorts, where sex-specific disparities may be more pronounced. Additionally, there is a clear need for more nuanced and standardized outcome parameters to enhance our understanding of sex differences in functional recovery after MT.

### 4.3. Sex Dependent Associations of Prognostic Markers with Outcome

To date, there is limited evidence on whether specific clinical or imaging markers have sex-specific prognostic value for functional outcomes after MT. Our findings suggest that several widely used predictors may exhibit sex-dependent associations. In our cohort, core lesion volume emerged as a sex-specific imaging marker, being significantly linked to favorable outcomes in men. A similar sex-specific association was reported by Wróbel et al., although in their study, core lesion volume was connected to unfavorable outcomes in women [17]. In contrast, among female patients in our study, baseline NIHSS and the TICI score were significantly associated with favorable outcomes, both at discharge and at 90 days. These findings are consistent with a subgroup analysis by Sun et al., which found NIHSS to be prognostically relevant in women with milder strokes [27]. The observed association between better angiographic recanalization results and functional outcome in women highlights the particular importance of successful reperfusion in this group. The association we observed between a higher penumbra volume and a worse outcome in women fits with the findings of Lagebrant et al. and Wróbel et al. [17,19] and may indicate that the ischemic tissue at risk is more vulnerable in women than in men. These sex-specific differences in prognostic relevance may have important implications for the development of individualized predictive models and tailored post-stroke care. They emphasize the need to systematically consider biological sex as a modifying factor in future studies and prognostic analyses. The underlying mechanisms driving these differences remain unclear and may involve social, healthcare system–related or biological factors, such as sex-specific functional cerebral asymmetries [37]. Further research is warranted to elucidate sex-related differences in stroke recovery and to support the advancement of precision medicine through the identification of robust, sex-sensitive prognostic biomarkers.

### 4.4. Limitations and Future Directions

This study has several limitations. First, its retrospective design inherently introduces the potential for bias and limits causal inference. Second, the moderate sample size resulting from the single-center design may reduce generalizability. However, the monocentric setting ensured consistent diagnostic and therapeutic standards across all cases. Moreover, the analysis focused solely on standard clinical and imaging parameters routinely available in acute stroke care. Additional prognostic factors—such as lesion water uptake, involvement of white matter tracts, or premorbid functional status (e.g., premorbid mRS)—were not included, although they may influence outcomes and interact with sex-specific recovery patterns. The use of the mRS as the primary outcome measure, while widely accepted, may not be sophisticated enough to capture subtle differences in functional recovery between sexes. Finally, the potential for selection bias in single-center data underscores the need for future multicenter studies with larger, more diverse cohorts and more refined outcome measures to validate and expand upon these findings.

## 5. Conclusions

Our findings indicate that women and men are equally likely to achieve favorable outcomes after MT for LVO. However, women showed less favorable results when considering the full range of disability. We identified distinct sex-specific prognostic markers, with NIHSS, TICI score and penumbra volume associated with outcomes in women, while core lesion extent was associated in men. Age emerged as the only consistent prognostic factor across both sexes. These results underscore the importance of considering sex differences when analyzing outcomes, developing predictive models, and designing clinical trials. Future studies should include sex-stratified analyses and integrate more detailed outcome measures to better understand and address sex-specific recovery trajectories after stroke.

## Figures and Tables

**Table 1:** Patient Baseline Clinical and Demographic Characteristics.

Variable	n = 183	Male (n = 91)	Female (n = 92)	*p*-Value
Age (±SD) in yrs	72 ± 13	70 ± 13	75 ± 12	* 0.005
Median NIHSS (IQR)	16 (11–18)	15 (11.5–18)	16 (11–18)	0.623
Median ASPECTS (IQR)	7 (6–9)	7 (5.5–8)	8 (6.75–9)	0.086
Core lesion (±SD) (in % of whole brain vol.)	7 (5)	6 (4)	7 (6)	0.366
Core lesion (±SD) in mL	70 (50)	70 (45)	70 (55)	0.981
Median collateral status (IQR)	2 (2–3)	2 (2–2)	2 (2–3)	0.155
Time symptom to admission (min ± SD) (n = 118)	96 (60)	92 (59)	99 (68)	0.554
Time imaging to reperfusion (min ± SD) (n = 170)	114 (50)	109 (43)	122 (56)	0.111
Number (proportion%) of patients with				
affection of the right hemisphere	93 (51)	47 (52)	46 (50)	0.825
IV thrombolysis received	125 (68)	63 (69)	62 (67)	0.791
Successful recanalization (≥TICI 2b)	113 (62)	61 (67)	52 (57)	0.145
Vessel occlusion in CT angiography				
Proximal ICA	50 (27)	29 (32)	21 (23)	0.186
Distal ICA	15 (8)	9 (10)	6 (7)	0.422
Distal ICA/carotid T	22 (12)	12 (13)	10 (11)	0.652
M1	101 (55)	46 (51)	55 (60)	0.181
Diagnosis of				
Arterial hypertension	120 (66)	59 (65)	61 (66)	0.836
Diabetes mellitus	40 (22)	18 (20)	22 (24)	0.615
Hypercholesterolemia	46 (25)	23 (25)	23 (25)	0.967
Atrial fibrillation	80 (44)	32 (35)	48 (52)	* 0.021
History of ischemic stroke	17 (9)	6 (7)	11 (12)	0.214

SD standard deviation; IQR interquartile range; IV intravenous; TICI Thrombolysis in Cerebral Infarction score; ICA internal carotid artery, CT computed tomography; Asterisks highlight statistically significant values.

## Data Availability

The data presented in this study are available upon reasonable request.

## Supplementary Materials

The following supporting information can be downloaded at: https://www.mdpi.com/article/10.3390/jcm14217855/s1, Figure S1: Association between sex and functional outcome.

## Abbreviations

The following abbreviations are used in this manuscript:

LVO	Large vessel occlusion
MT	Mechanical thrombectomy
ASPECTS	Alberta stroke program early CT score
NIHSS	National Institutes of Health Stroke Scale
mRS	Modified Rankin scale
RCT	Randomized controlled trials
NCCT	Non-enhanced cranial CT
CBF	Cerebral blood flow
Tmax	Time-to-maximum of the tissue residue function
MCA	Middle cerebral artery
TICI	Thrombolysis in cerebral infarction
OR	Odds ratio
CI	Confidence interval
IQR	Interquartile range
SD	Standard deviation
ICA	Internal carotid artery
