# Supplementary material for: Sex Differences in Prognostic Markers: Exploring Outcome Variability After Mechanical Thrombectomy in Large Vessel Occlusion Stroke
Source: J Clin Med. 2025 Nov 5;14(21):7855. doi: 10.3390/jcm14217855 (PMC12608539; doi:10.3390/jcm14217855)
Supplement: Supplementary file 1 [file jcm-14-07855-s001.zip › jcm-3912729-supplementary.pdf]

Supplementary Online Material

Sex Differences in Prognostic Markers: Exploring Outcome Variability after Mechanical Thrombectomy in Large Vessel Occlusion Stroke

Hannes Schacht [1,*], Alexander Neumann [1], Nora Petersen [2], Lis Merrit Ehm [2], Maria Marburg [2], Christine Matthis [3], Ulf Jensen-Kondering [1], Peter Schramm [1], Jens Minnerup [2], Georg Royl [2] and Philipp J. Koch [2,4]

1 Department of Neuroradiology, University Hospital Schleswig-Holstein, Campus Lübeck, 23538 Lübeck, Germany; Alexander.Neumann@uksh.de (A.N.); Ulf.Jensen-Kondering@uksh.de (U.J.-K.); Peter.Schramm@uksh.de (P.S.)
2 Department of Neurology, University Hospital Schleswig-Holstein, Campus Lübeck, 23538 Lübeck, Germany; nora.petersen@student.uni-luebeck.de (N.P.); LisMerrit.Ehm@uksh.de (L.M.E.); MariaJoana.Marburg2@uksh.de (M.M.); Jens.Minnerup@uksh.de (J.M.); Georg.Royl@uksh.de (G.R.)
3 Department of Social Medicine and Epidemiology, University Hospital Schleswig-Holstein, Campus Lübeck, 23538 Lübeck, Germany; Christine.Matthis@uksh.de (C.M.)
4 Department of Neurology and Experimental Neurology, Charité - University Medicine Berlin, corporate member of Freie Universität Berlin and Humboldt-Universität zu Berlin, 10117 Berlin, Germany; philipp-johannes.koch@charite.de (P.J.K.)
* Correspondence: Hannes.Schacht@uksh.de; Tel.: +49-45150076183

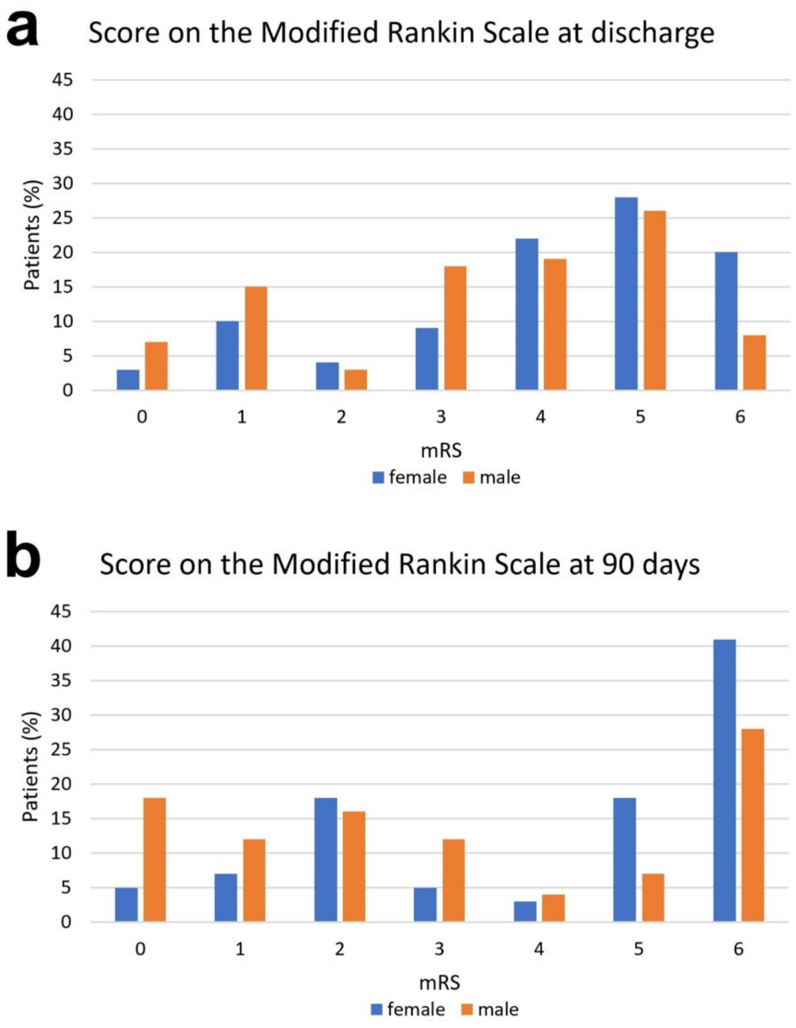

Figure S1. Bar graphs illustrate the overall differences between female and male patients in the modified Rankin Scale (mRS) at discharge (a) and 90 days following stroke (b).
